# Mammalian cellular culture models of *Trypanosoma cruzi* infection: a review of the published literature

**DOI:** 10.1051/parasite/2014040

**Published:** 2014-08-04

**Authors:** Gabriel Alberto Duran-Rehbein, Juan Camilo Vargas-Zambrano, Adriana Cuéllar, Concepción Judith Puerta, John Mario Gonzalez

**Affiliations:** 1 Grupo de Ciencias Básicas Médicas, Facultad de Medicina, Universidad de los Andes Bogotá, DC Colombia; 2 Grupo de Inmunobiología y Biología Celular, Facultad de Ciencias, Pontificia Universidad Javeriana Bogotá, DC Colombia; 3 Laboratorio de Parasitología Molecular, Facultad de Ciencias, Pontificia Universidad Javeriana Bogotá DC Colombia

**Keywords:** *Trypanosoma cruzi*, *In vitro* cell culture, Chagas disease, Literature review

## Abstract

Cellular culture infection with *Trypanosoma cruzi* is a tool used to dissect the biological mechanisms behind Chagas disease as well as to screen potential trypanocidal compounds. Data on these models are highly heterogeneous, which represents a challenge when attempting to compare different studies. The purpose of this review is to provide an overview of the cell culture infectivity assays performed to date. Scientific journal databases were searched for articles in which cultured cells were infected with any *Trypanosoma cruzi* strain or isolate regardless of the study’s goal. From these articles the cell type, parasite genotype, culture conditions and infectivity results were extracted. This review represents an initial step toward the unification of infectivity model data. Important differences were detected when comparing the pathophysiology of Chagas disease with the experimental conditions used in the analyzed studies. While *Trypanosoma cruzi* preferentially infects stromal cells *in vivo*, most of the assays employ epithelial cell lines. Furthermore, the most commonly used parasite strain (Tulahuen-TcVI) is associated with chagasic cardiomyopathy only in the Southern Cone of South America. Suggestions to overcome these discrepancies include the use of stromal cell lines and parasite genotypes associated with the known characteristics of the natural history of Chagas disease.

## Abstract (fr)

L’infection de cultures cellulaires avec *Trypanosoma cruzi* est un outil utilisé pour disséquer les mécanismes biologiques à l’origine de la maladie de Chagas ainsi que pour cribler des composés trypanocides potentiels. Les données sur ces modèles sont très hétérogènes, ce qui représente un défi pour comparer les différentes études. Le but de cette synthèse est de fournir un aperçu des essais d’infectivité de cultures cellulaires réalisés à ce jour. On a recherché dans les bases de données de revues scientifiques les articles dans lesquels des cellules en culture ont été infectées par n’importe quelle souche ou isolat de *Trypanosoma cruzi*, quel que soit l’objectif de l’étude. Le type de cellules, le génotype du parasite, les conditions de culture et les résultats d’infectiosité ont été extraits. Cette synthèse représente une première étape vers l’unification des données du modèle d’infectiosité. Des différences importantes ont été détectées lors de la comparaison de la physiopathologie de la maladie de Chagas avec les conditions expérimentales utilisées dans les études analysées. Bien que *Trypanosoma cruzi* infecte de préférence les cellules stromales *in vivo*, la plupart des essais emploient des lignées de cellules épithéliales. En outre, la souche la plus couramment utilisée de parasite (Tulahuen-TcVI) est associée à une myocardiopathie chagasique seulement dans le cône sud de l’Amérique du Sud. Des suggestions pour surmonter ces divergences comprennent l’utilisation de lignées de cellules stromales et des génotypes parasitaires associés aux caractéristiques connues de l’histoire naturelle de la maladie de Chagas.

## Introduction

Chagas disease is a complex parasitic infection caused by *Trypanosoma cruzi*, which is influenced by a wide variety of factors that affect its natural history: (a) an overwhelming amount of animal reservoirs which make parasite eradication virtually impossible, (b) poor housing conditions in rural endemic areas which facilitate vector reproduction and parasite transmission, (c) difficulty in diagnosis due to the unspecific, usually subclinical presentation of the acute form and the long evolution of the chronic phase, (d) lack of pharmacological and immunological prophylactic measures (i.e. human vaccines), and (e) existence of only two compounds (benznidazole and nifurtimox) for antiparasitic therapy [4, 29].

*Trypanosoma cruzi* is a hemoflagellate protozoan transmitted to humans by arthropods of the subfamily Triatominae, mostly confined to Latin America. Triatomines are hematophage insects that ingest the trypomastigotes found in infected host blood while feeding. After a series of parasite changes inside the vector’s digestive tract, the infective forms of the parasite (metacyclic trypomastigotes) are ejected along with the insect’s stools. Metacyclic trypomastigotes enter mammalian host cells via breaches of the skin or through mucosae such as the conjunctiva [23]. Other forms of transmission include blood transfusion, vertical transmission, laboratory accidents, and oral infection by the ingestion of food contaminated with insect stools [4]. The parasites replicate as amastigotes inside several cells including monocytes/macrophages and dendritic cells [7, 29], ultimately entering the bloodstream as trypomastigotes where they can spread to and potentially infect virtually any human nucleated cell. The illness has two major clinical forms: an acute and a chronic phase. During the acute phase, usually days after parasite inoculation, symptoms are unspecific and include fever, malaise, reactive lymphadenopathy, and subcutaneous edema [23]. After recovery from the acute infection following a competent immune response, all patients enter the indeterminate form of chronic Chagas disease characterized by a lack of symptoms and a positive serological test for anti-*T. cruzi* antibodies [1]. Approximately one-third of indeterminate individuals progress to the symptomatic or determinate form of the chronic phase, which consists of abnormalities in the cardiovascular (conduction system alterations, arrhythmias, and dilated cardiomyopathy which can result in heart failure and death) or gastrointestinal (alterations in esophageal and colonic motility) systems [23, 29]. In both cases, the mechanisms of pathogenesis have been associated with parasite persistence, certain parasite genotypes and an antigen-induced dysfunction of the immune response [9, 15, 29].

*Trypanosoma cruzi* is currently classified into six Discrete Typing Units (DTUs) based on genomic sequencing: TcI through TcVI [38]. Although currently the subject of ongoing studies, it is hypothesized that TcI strains are more commonly found in cardiac involvement, while the TcII-V-VI complex isolates are more commonly associated with gastrointestinal disease [39].

One of the tools used to unravel the mechanisms of *T. cruzi* infection such as cellular tropism, infective capacity, and intracellular reproduction is the infection of *in vitro* cell cultures. Ever since the first report of *in vitro* infection using human cardiomyocytes [11] and cell lines [6], Vero (renal epithelial cells from the African green monkey) and HeLa (human cervical cancer-derived cells) have been some of the eukaryotic cell lines used as infection models [5, 18]. Mammalian cell cultures are used for a number of purposes which include propagating trypomastigotes from other cell culture types or animals [21], testing trypanocidal compounds in order to determine if a particular molecule is able to kill the parasite without damaging the host cell [11] and notably to study the host-parasite interaction as well as to examine potential putative receptors for *T. cruzi* [7]. Currently, there are a wide variety of cell lines, parasite strains, and culture conditions in use. This diversity, while reflecting the ever-increasing expansion of knowledge with regard to parasite-host infection models, also represents an obstacle when it comes to comparing the different experimental conditions. In consequence, it would be very useful to have an outline of the different methodologies currently used for parasite culture. With this in mind, the main purpose of this article is to present an overview of infection assays with *T. cruzi*-mammalian cell culture to get a better understanding of parasite infection characteristics and methodologies.

## Materials and methods

### Searching strategy for databases

Scopus, ISI Web of Knowledge, PubMed and LILACS were searched for the following MeSH terms for English and Decs terms for Spanish and Portuguese: “*Trypanosoma cruzi* or *T. cruzi*” and “cell culture”. Specific searches were conducted in each database for human-, bovine-, and primate-derived cell lines. Searches were conducted for articles published up to March 2013. The study followed the requirements of the PRISMA statement (www.prisma-statement.org); see the supporting information checklist and flowchart.

### Selection criteria of articles and selection process

The following criteria were applied for selection: (a) articles written in English, Portuguese, or Spanish, (b) experiments involving any *Trypanosoma cruzi* strain, (c) *in vitro* experiments including cell infection assays in their methodology regardless of the objective pursued, and (d) experiments using human-, bovine-, or primate-derived cell lines. Articles that met all four criteria were included. Each article was carefully reviewed by at least two independent readers (GD, JCV or JMG) in order to extract data regarding the infection models.

### Data extracted from selected articles

The following data were extracted from the selected studies: cell line type, *T. cruzi* strain or isolate, multiplicity of infection (MOI, defined as the ratio of parasites to host cells), cell-parasite incubation time, general culture conditions (media, serum supplementation, and percentage of serum), infectivity results (given as percentage of infected cells, number of intracellular amastigotes per cell or number of amastigotes per hundred cells), and type of study conducted. Where MOI values were not stated, they were calculated or approximated according to the information given in each article. Studies were classified into one of the following general categories: immunological (defined as studies evaluating any aspect of the immune response elicited by *T. cruzi* infection), pharmacological (studies evaluating the potential trypanocidal or growth-inhibitory effect of natural or synthetic compounds), or biological (studies focusing on the parasite’s biology). Biological studies were further sub-classified into one of three sub-categories: morphological, infectivity (attachment, cellular invasion, or reproduction), or biochemical studies. *T. cruzi* nomenclature was updated to current standards as defined by Zingales et al. when possible [38].

## Results

### Literature search

**Figure 1.**
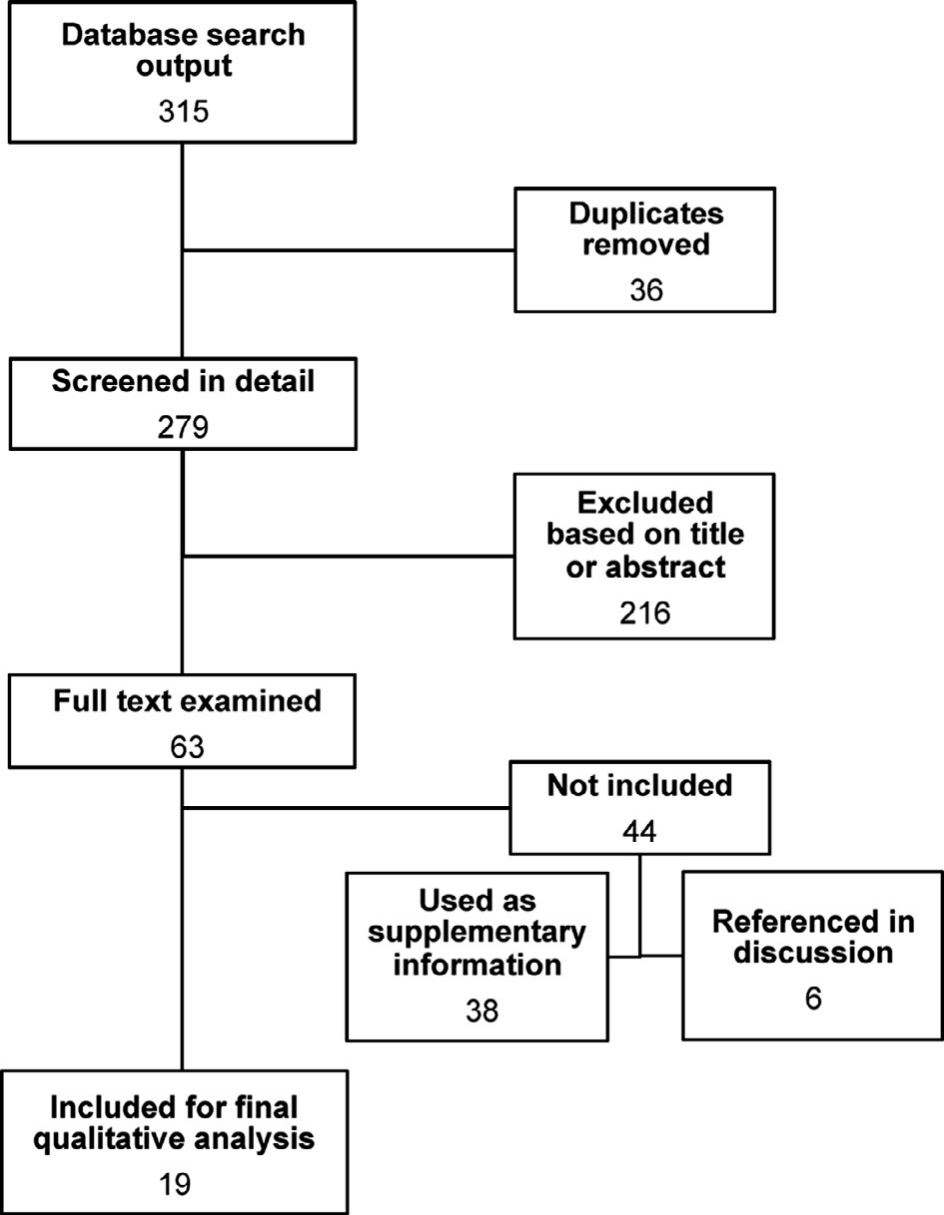
Flow diagram for selected articles from databases.

Using the methodology described above (Fig. 1), a total of 315 articles were obtained, of which 36 were discarded for duplication (11% redundancy), and 279 were screened in detail. After a second round of revision 216 articles were removed because they did not fully meet the selection criteria. During this phase, the main criterion for removal of articles was information in the title or abstract that characterized the study as mutually exclusive to the selection criteria (i.e., experimental animal infection from which cells were later extracted or analysis of trypomastigotes derived from cell cultures rather than experimental infection assays). The final count of selected articles was 63 out of 279 (22.6%). Of these articles, 35 out of the 194 manuscripts (18.0%) were found in Scopus, 22 out of the 48 (45.8%) in PubMed, 2 out of the 8 (25.0%) in ISI Web of Knowledge, and 4 out of the 29 (13.8%) in LILACS.

After review by at least two independent readers, 38 of these 63 (60.3%) were further excluded because they did not present infectivity assay data despite including infection procedures in their methodology or because they were found to not fully meet the selection criteria (review articles or use of tissue samples instead of cell lines). Despite not presenting infectivity data, important information was found in these manuscripts (*n* = 38, see Supporting Table I) as follows: (a) the most commonly used cell lines were Vero (African green monkey renal epithelial cells; *n* = 7), LLC-MK2 (Rhesus monkey kidney epithelial cells; *n* = 7), peripheral blood mononuclear cells (PBMCs; *n* = 6), and human placenta derivatives (*n* = 3), among others, (b) the most common parasite strains used were Tulahuen (TcVI; *n* = 16), Y strain (TcII; *n* = 8) and Brazil (not classified into DTU; *n* = 2); and (c) immunological (*n* = 14) and biological (*n* = 14) studies were the most frequent type of study. Biochemical studies (*n* = 9) represent the majority of these studies. Although not originally considered, because of the amount of articles found which used PBMCs, it was decided to take these studies into account for the final analysis.

Of the 25 remaining articles, six did not fully meet the selection criteria, but contain important information in their methodologies such as: the effects of the incubation temperature on the life cycle of *T. cruzi* [6], *in vitro* parasite division time [14], differential attractiveness of mammalian cell lines to the parasite [5], parasite fluorescent gene transfection for culture infection [10], a recount of the use of different cell lines for infectivity assays with *T. cruzi* [25], and one article discussing the role of calcium metabolism in parasite infectivity [37]. Consequently, they were not included in Table 1 but will be referenced in the Discussion section.

**Table 1.** Summary of data extracted from articles which met the selection criteria.

Reference	Cell line	*T. cruzi* strain (DTU)	MOI	Incubation time	Medium (Supplement.)	Results	Type of study
Osuna et al., 1990 [19]	HeLa	Human isolate (NT)	1, 5 & 10	2 h	MEM (ND)	% of infection % PI 2 h MOI 1 (21.9 & ND) MOI 5 (35.5 & 1.6) MOI 10 (20.2 & 2.2)	Biological: biochemistry
Faria et al., 2008 [8]	HeLa	Y (TcII)	40	0, 1, 3, 6, 12 & 24 h	DMEM (10% FCS)	% of infection at hrs: 1 (>20%), 3 (>40%), 6 (<50%), 12 (>50%) & 24 (>80%)	Biological: biochemistry
Sartori et al., 2003 [26]	HEp-2	Tulahuen (TcVI)	10	2 h	MEM (10% FCS)	% of infection 60%–80%	Biological: infectivity
Morris et al., 1988 [16]	HUVEC	Tulahuen (TcVI)	1.5–2	4 d	M199 (20% FCS 10% HS)	% of infection at 4 days: 40–50%. In 75% of the cells NoA of at least 5	Biological: biochemistry
Wittner et al., 1995 [36]	HUVEC & HUSMC	Tulahuen (TcVI)	1.5 & 2	72 h	ND (ND)	% of infection at hrs: 1 (<1%), 6 (<1%), 24 (10%), 48 (20–40%) & 72 (>80%)	Biological: biochemistry
Todorov et al., 2003 [30]	HUVEC	Dm28c (TcI)	1 & 4	3 h – wash & – 3 d	M199 (10% FCS)	NoA/100 cells at 3 hrs < 6. NoA/100 cells at 3d 298.4	Biological: infectivity
Mukherjee et al., 2004 [17]	HUVEC & HUSMC	Tulahuen (TcVI)	1.5 & 2	48 h – wash – 24 h	DMEM (20% FCS 5% HS)	% of infection at hrs: 24 (20%) 48 (50%) & 72 (80%)	Biological: biochemistry
Hassan et al., 2006 [12]	HUVEC & HUSMC	Tulahuen & Brazil (TcVI & NT)	1.5 & 2	48 h – wash – 24 h	ND (ND)	% of infection at hrs: 24 (20%), 48 (50%) & 72 (80%)	Biological: biochemistry
Soares et al., 2011 [27]	PBMCs	Y (TcII)	10	3 h – wash & add compound – 24 h	RPMI (10% FCS)	Mean % of infection & NoA/infected cell from 8 human donors: 32% ± 21,48% & 2,61 ± 1,61	Pharmacological
Souza et al., 2007 [28]	PBMCs	Y (TcII)	10	3 h	RPMI (ND)	% of infection at 3 h: monocytes (80%), T-lymphocytes (1%) & B-lymphocytes (5%)	Immunological
Williams and Remington, 1977 [35]	PBMCs	Tulahuen (TcVI)	1	2, 24, 48 & 72 h	M199 (40% HS)	% of infection monocytes: hrs 2 (29%), 24 (25%) & 48 (26%). Macrophages: average % of infection 90% at all time points.	Biological: infectivity
Coelho dos Santos et al., 2010 [2]	PBMCs	Y (TcII)	5	3, 48 & 96 h	RPMI (10% FCS)	% of infection at hrs: 3 (>50%), 48 (25–50%) & 96 (25–50%)	Immunological
Piras et al., 1982 [21]	Vero	EP, BEC, MEN (NT, NT & NT)	NS	2 h	MEM (10% FCS)	Ii: EP strain 0.8; BEC strain 0.19.	Biological: morphology
Piras et al., 1987 [20]	Vero	EP (NT)	ND	2 h	MEM (10% FCS)	Ii: medium alone 0.4, with FCS 1.0	Biological: infectivity
Urbina et al., 1988 [31]	Vero	EP (NT)	20	2 h – wash – 100 to 180 h	MEM (10% FCS)	% of infection at hrs 2 (65%) 24 (69%), 48 (70%), 72 (70%), 96 (85%). Mean NoA/cell: 60 at 96 h	Pharmacological
Urbina et al., 2002 [32]	Vero	EP (NT)	10	2 h – wash – 96 h	MEM (1% FCS)	% of infection & NoA/cell at 96 h: 23% & 30	Pharmacological
Revollo et al., 1998 [24]	Vero	Group 19/20: SP104 cl1, Cutia cl4, Gamba, 13379 cl7, OPS21 cl11, SO34 cl4, Cuica cl1, P/209 cl1, Esquilo cl1 & P/11 cl2 (TcI)	16	15–30 h	RPMI (5% FCS)	% of infection & NoA Group 19/20: 86,63 ± 7,89 & 17,98 ± 3,02	Biological: biochemistry
		Group 32: MAS1 cl1, CBB cl3, Tu18 cl2, IVV cl4 & MVB cl8 (TcV)				% of infection & NoA Group 32: 71,46 ± 7,25 & 13,67 ± 2,11	
		Group 39:SC43 cl1, Bug2148 cl1, Bug2149 cl1 & SO3cl5 (TcII)				% of infection & NoA Group 39: 51,27 ± 8,56 & 10,6 ± 2,37	
Pires et al., 2008 [22]	Vero	CL Brener, Tulahuén, JG & Col1.7G2 (TcVI, TcVI, TcII & TcI)	10	18 h – change medium – 6 d	LIT (ND)	% of infection & NoA/infected cell at 6d: TulaWT (wild-type) 7% & 25, TulaRFP1 9% & 20, TulaGFP2 7% & 30.	Biological: infectivity
Vilchez-Larrea et al., 2012 [34]	Vero	CL Brener (TcVI)	50	24 h – change medium – 5 d	DMEM (10% FCS)	% of infection & NoA: days 2 (37.20% & < 1), 4 (20.81% & < 3), 6 (27.36% & < 4)	Pharmacological

d: Days; DTU: Discrete typing unit; Ii:Infection index; FCS: Fetal calf serum; h: Hours; HS: Human serum; Ii: Infection index; MOI: Multiplicity of infection; PI: Parasite index; ND: No data; NoA: Number of amastigotes; NT: Not typified; PBMCs: Peripheral blood mononuclear cells; Tc: *Trypanosoma cruzi* DTU.

### Findings

Of the final selection of articles (*n* = 19), infectivity data results were presented in one of three ways: percentage of infected cells, number of amastigotes per infected cell [referred to as the parasite index (Pi) or infectivity index (Ii) as defined by some authors] or number of amastigotes per one hundred cells [19, 20, 24] (Table 1). The infectivity values were mostly obtained using light microscopy and Giemsa staining. Remarkably, one study used CFSE staining and flow cytometry for the parasite infection count [22]. Thirteen of these articles were biological studies, being mostly biochemical (*n* = 7) or infectivity (*n* = 5) studies. The most commonly used cell line was Vero (*n* = 7), followed by HUVEC (human umbilical vein endothelial cells; *n* = 5), PBMC (*n* = 4) and HeLa (*n* = 3). The *T. cruzi* strain most frequently employed was Tulahuen (TcVI; *n* = 7), followed by the Y strain (TcII; *n* = 4) and EP isolate (not classified into DTU; *n* = 4). MOI, taken directly or calculated from all studies, ranged from 1 [35] to 50 parasites per host cell [34]. Parasite-cell incubation times were widely spread, from 2 h [19] to as long as 6 days [22]. The culture media most frequently employed were MEM or DMEM (*n* = 11), RPMI (*n* = 4), M199 (*n* = 3), and LIT (*n* = 1). Two articles did not report the medium used; but referred to manuscripts where DMEM was utilized. Fifteen works reported the use of media supplementation with fetal calf serum (FCS) in concentrations ranging from 1% to 20% (*n* = 14). Human serum (HS) was used in three studies at concentrations of 5%, 10%, and 40%, respectively. Two articles report having used both FCS and HS medium supplementation simultaneously – Table 1 [16, 17].

Experiments using PBMCs incubated the cells with trypomastigotes for short periods of time (<3 h), indicating that the parasite can be rapidly detected within monocytes. After 2–3 h of incubation, the percentage of parasitized monocytes ranged from 29% to 80% [2, 28, 35]. Macrophages incubated for the same time period showed a consistently higher percentage of intracellular parasites with nearly 90% infection at all time points from 2 to 48 h [35]. There is one result regarding lymphocyte infection in studies using PBMCs [28]. In a similar fashion to primary human monocytes/macrophages, HeLa cells and derivatives (HEp-2) were highly susceptible to *T. cruzi* infection (80% of infected cells at 24 h) regardless of the parasite strain or MOI used [8, 19, 26]. Similar percentages of infected cells (approximately 80%) were observed when HUVEC or HUSMC (human umbilical smooth muscle cells) cells were used. However, parasites required longer periods of time (72 h) to grow and complete their development in these latter cells [12, 30, 36].

In Vero cells, infectivity results tend to vary with the parasite strain or isolate used. Indeed, the EP isolate (not classified into DTU) produces greater infectivity than CL Brener (TcVI) and Tulahuen (TcVI) – Table 1. After 96 h of parasite-cell incubation with EP, 23–85% of cells were infected and contained 30–60 amastigotes per infected cell, with values varying according to the MOI and FCS concentration used [31, 32]. When the MOI and FCS concentration were increased, infectivity was enhanced. In contrast, after 96 h of incubation, CL Brener-infected cells displayed 20.8% infection and less than three amastigotes per cell despite using a MOI which is 10 times higher than in experiments with EP [34]. Furthermore, infectivity in Vero cells with the EP isolate was higher than that seen with BEC isolate when using identical conditions [21]. These observations are consistent with a report by Revollo et al., which highlights the repercussions of parasite genetics on infectivity in Vero cells [24]. In this report several stocks of the parasite were classified into one of three groups based on genomic analysis. Group 19/20 (mostly TcI) averaged 86% infection, group 32 (mostly TcII) averaged 71%, and group 39 (mostly TcV) averaged 51% [24]. This difference of up to 35% infectivity across groups of *T. cruzi* stocks treated otherwise identically suggests that besides MOI and culture conditions, the parasite genotype is also a crucial factor for infection of epithelial cells such as Vero. Based on these results, infectivity for TcI and TcII *T. cruzi* genotypes was similar to that presented for the EP isolate, while infection with TcV strains was lower.

**Figure 2.**
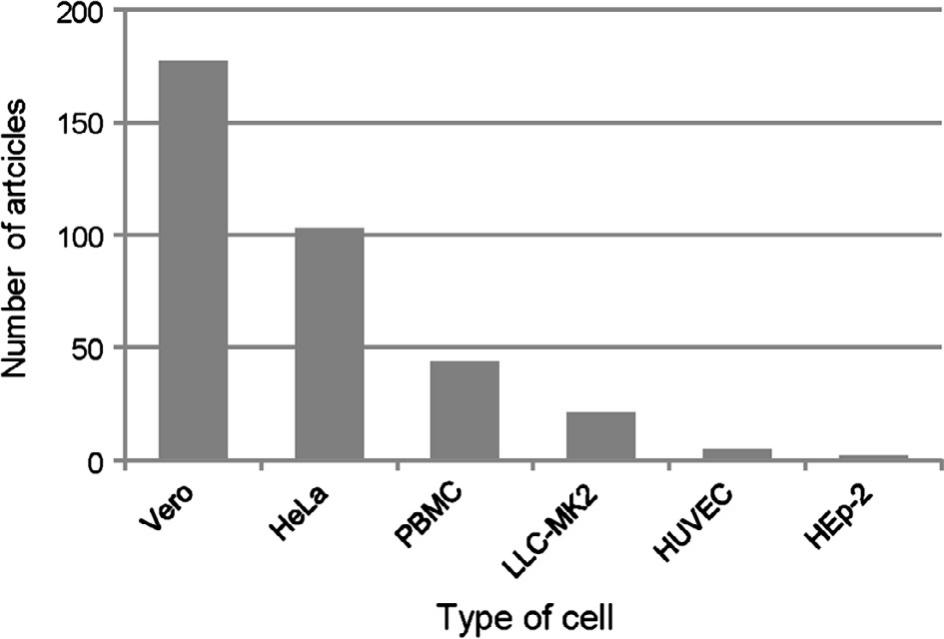
Number of articles found searching for *Trypanosoma cruzi* and specific cell lines in www.pubmed.org. Each bar represents the number of articles found until April 2013 when searching PubMed for: Vero, PBMCs (peripheral blood mononuclear cells), HeLa, LLC-MK2, HUVEC (human umbilical vein endothelial cells), and HEp-2 (HeLa derivative).

The results presented here can be considered a sampling of the literature with regard to *T. cruzi* cell line infection assays. An independent search was conducted in PubMed with the MeSH terms “*Trypanosoma cruzi*” or “*T. cruzi*”, and each of the cell types mentioned. The results produced 177 articles for Vero cells, 103 for HeLa, 44 for PBMCs, 21 for LLC-MK2, 5 for HUVEC, and 2 for HEp-2 – Figure 2. The four most commonly employed cell lines found in the literature search are also the four most commonly used cell lines found with our methodology when pooling articles included for the final analysis and those excluded because of incomplete infectivity data. This fact demonstrates that the results of this review reflect the wide array of articles published.

## Discussion

Given the intracellular nature of *T. cruzi* in its mammalian hosts, cellular infection assays are an essential tool to dissect the pathogenesis of Chagas disease. They allow for an approximation to study the dynamics of parasite-host cell interactions [37]. Nonetheless, the methodologies of these assays display great variability, which continues to increase as the number of articles published on this topic grows. This leads to a highly heterogeneous amount of information, which can be difficult to compare. Consequently, the goal of this study is to present an overview of *T. cruz*i cell line infectivity assays specifically in terms of cell types, parasite strains, culture conditions, and infectivity results. The results obtained, as well as the relevant information extracted from excluded articles, will be discussed based on the order in which the events of *Trypanosoma cruzi* infection occur.

After finding a host cell, *T. cruzi* interacts with its putative extracellular receptors, triggering a phosphorylation cascade that ultimately culminates in the increase in intracellular calcium, promoting trypomastigote internalization [37]. Parasite attachment and infectivity of cell lines is enhanced by the presence of serum, as the infection index on Vero cell culture increased from 0.4 in medium alone to 1.0 in the presence of FCS. This supports the notion that serum provides factors such as sialoglycoproteins that are needed for parasite attachment and penetration of host cells [20]. Temperature has also been described as a determining factor for both host cell internalization and intracellular reproduction. Parasite reproduction (defined as the time elapsed between parasite internalization and division) is faster at 37 °C than at lower temperatures. The “doubling time” or the period required for the division of amastigotes is shorter at 37 °C than in colder environments [6].

Another factor that has been known to influence parasite infectivity is the type of host cell it is maintained in. Parasites kept exclusively in BESM (bovine embryo skeletal muscle) cell culture showed longer duplication times than parasites passed through insect vectors, indicating that the longer a *T. cruzi* strain or isolate is kept without passing through its intermediate host, the less virulent it becomes [14]. Notably, parasite infectivity was also unaffected after transfection with a fluorescent marker in cultures on Vero [22], LLC-MK2 cells and even in the insect vector [10].

Within the published literature, two of the most commonly used cell lines (Vero and LLC-MK2) are both renal epithelial cells derived from primates. The human cervical epithelial carcinoma HeLa cells and HUVEC (endothelial) are frequently used as well, indicating that current research with *T. cruzi* infection models relies heavily upon the use of epithelial cells. In contrast, during *in vivo* acute infection, metacyclic trypomastigotes seem to have a predilection for stromal cells of the cardiovascular, reticuloendothelial, nervous, and muscular systems [13]. Because of this, it could be argued that the use of cell lines derived from a similar stromal origin would allow for an *in vitro* study of infection that more closely resembles events *in vivo*. Furthermore, in models such as those employed in pharmacological studies, the use of culture cell lines, which are actively dividing (such as VERO or HeLa), instead of primary cells may modify output data regarding the toxicity and specificity of the tested antiprotozoal agents. Our group has recently used a new human cell line derived from a glioblastoma multiforme with the aim of determining if astrocytes can harbor *T. cruzi* (TcI) as a model for CNS infection [33].

Non-epithelial cells such as monocytes/macrophages are highly parasitized after very short incubation times, providing insight into the initial stages of *T. cruzi* infection and the subsequent immune response. Taking into account the parasite-induced dysfunction of the immune response as a key element in the pathogenesis of chronic Chagas disease, it is crucial to further study the interaction between *T. cruzi* and other cells of the immune system such as PBMCs. Furthermore, data on other immune cells in relation to *T. cruzi* infection such as T-lymphocytes would contribute to the understanding of the pathogenesis of Chagas disease [9].

In this review, Tulahuen (TcVI) was by far the most commonly used *T. cruzi* strain, followed by the Y (TcII) strain. A recent update on the epidemiology of *T. cruzi* and the clinical implications of parasite DTUs indicated that: (a) TcI is the most common DTU associated with acute Chagas disease, (b) TcI is the DTU most frequently found in chronic Chagasic patients with cardiomyopathy from the northern countries of South America, and (c) TcII-TcV-TcVI DTUs are the most commonly isolated from Chagasic patients with cardiomyopathy from the Southern Cone [39]. Moreover, TcI is also the DTU most frequently isolated from immune-compromised individuals with reactivation of Chagas disease [3]. Overall, these findings highlight the need to conduct more studies using TcI strains.

*In vitro* cellular infection models represent a tool for the study of Chagas disease. Further research is required in order to widen the existing knowledge on the pathophysiology of Chagas disease and potential therapeutic targets. As this research develops, it would be ideal to employ stromal cell lines, which bear a closer resemblance to the targets of *in vivo* infection, as well as parasite strains/isolates associated with the acute or chronic form of the disease according to the scope of each study.
